# Hypoxic Hospitals After Happy Hypoxia During Coronavirus Disease 2019 Pandemic

**DOI:** 10.5152/TJAR.2021.20412

**Published:** 2022-04-01

**Authors:** Ankur Sharma, Varuna Vyas, Shilpa Goyal, Nikhil Kothari

**Affiliations:** 1 Department of Trauma & Emergency, All India Institute of Medical Sciences, Jodhpur, Rajasthan, India; 2 Department of Paediatrics, All India Institute of Medical Sciences, Jodhpur, Rajasthan, India

## Dear Editor,

The current novel coronavirus disease 2019 (COVID-19) pandemic is affecting the global human population, including resource-limited countries. Most patients present with marked arterial hypoxemia but with no corresponding symptoms of respiratory distress. This condition is referred to as silent or “happy” hypoxemia.^1^ In severe cases, death can result from acute respiratory distress syndrome and pneumonia.

Supplemental oxygen is the first crucial step for COVID-19 therapy, and appropriate respiratory support is vital for patient survival. In COVID-19 patients, the magnitude of hypoxemia is independently correlated with in-hospital mortality and is a significant indicator of the patient’s probability of needing admission to the intensive care unit.^2^

Patients suffering from COVID-19 frequently require high-flow oxygen using a high-flow nasal cannula (HFNC) and ventilators.^3,4^ Subsequently, oxygen demand started to grow after the COVID-19 pandemic, as oxygen is a necessity for these critically ill patients. 

There might not be oxygen manifold and pipelines in resource-limited settings and district-level hospitals. In these conditions, oxygen therapy may be given to critically ill patients through oxygen cylinders. Consumption of oxygen from a cylinder relies on the fraction of inspired oxygen (FiO_2_), minute ventilation (tidal volume × respiratory rate), compliance of the lungs, inspiratory: expiratory ratio, and configuration of the ventilator. If a patient on HFNC consumes 60 liters per minute of 100% oxygen, it would be 3600 L of oxygen needed per hour. It indicates that the medical oxygen demand of a critical COVID-19 patient is approximately 86 000 liters per day (3600 × 24 = 86 400) on an HFNC.

To use these cylinders, one should know the period over which the cylinder would last. The following example can be used to measure the amount of time an oxygen cylinder will supply oxygen to a critically ill patient. If oxygen consumption is 60 L min^-1^ and the FiO_2_ is 100%, the H-type cylinder^5^ can last for 6900/60, that is, 115 minutes or only 1.91 hours. It indicates that oxygen cylinders should be available in adequate quantities.

To conclude, in resource-poor settings, adequate oxygen is necessary for managing severe COVID-19 patients with hypoxemia. As coronavirus cases increase rapidly, these regions have been impacted by a substantial medical oxygen supply shortage. All hospitals with COVID-19 patients must ensure the availability of an appropriate oxygen supply.

## References

[1] Dhont S Derom E Van Braeckel E Depuydt P Lambrecht BN . The pathophysiology of 'happy' hypoxemia in COVID-19. Respir Res. 2020;21(1):198. 10.1186/s12931-020-01462-5) PMC7385717 32723327

[2] Mehta Y Chaudhry D Abraham OC et al. Critical care for COVID-19 affected patients: position statement of the Indian Society of Critical Care Medicine. Indian J Crit Care Med. 2020;24(4):222 241. 10.5005/jp-journals-10071-23395) 32565632 PMC7297240

[3] Calligaro GL Lalla U Audley G et al. The utility of high-flow nasal oxygen for severe COVID-19 pneumonia in a resource-constrained setting: a multi-center prospective observational study. EClinicalmedicine. 2020;28. 10.1016/j.eclinm.2020.100570) PMC7536126 33043285

[4] COVID 19 and the oxygen bottleneck. Bull World Health Organ. 2020;98(9):586 587. PMID: 33012857; PMCID: PMC7463186. 10.2471/BLT.20.020920) 33012857 PMC7463186

[5] Srivastava U Anaesthesia gas supply: gas cylinders. Indian J Anaesth. 2013;57(5):500 506. 10.4103/0019-5049.120147) 24249883 PMC3821267

